# Enhancing Blood Collection Processes: A Phlebotomy Clinic Improvement Project

**DOI:** 10.1192/bjo.2025.10461

**Published:** 2025-06-20

**Authors:** Joshua Wilkinson, Anna Sherratt, Eleanor Davies

**Affiliations:** Coventry and Warwickshire Partnership NHS Trust, Coventry, United Kingdom

## Abstract

**Aims:** To determine whether an in-house phlebotomy clinic would reduce the length of time taken for blood tests to be completed and results reviewed within the CAMHS Eating Disorder Service in Coventry and Warwickshire Partnership NHS Trust, which would reduce patient safety incidents caused by these delays.

**Methods:** Collation of baseline data which includes the timeframe in days between:

Blood tests being requested and blood tests being taken.

Blood tests being taken and blood results being reviewed.

Blood tests being requested and blood results being reviewed.

This baseline data has been displayed in a statistical process chart to show the current length of time taken for each of the above. The baseline data also shows which patients had abnormal blood results that required A&E attendance for urgent repeat testing. It also reviews the near misses that occur due to the length of time taken to review blood results.

Testing change ideas using Plan-Do-Study-Act cycles for each change idea:

Created a central list of all patients who have had blood tests requested.

Introduced standardised days for checking if the blood test has been taken and reviewing the results accordingly.

Increased the number of standardised days for checking if the blood test has been taken.

Development of an in-house phlebotomy clinic.

**Results:** The statistical process charts show that since introducing the change ideas, the overall mean for the timeframe between blood tests being taken and blood results being reviewed has reduced from 17 days to 2 days. This shows the change in process has had a positive impact on this step. However, the same improvement has not been shown in the timeframe between blood tests being requested and blood tests being taken, nor blood tests being requested and blood results being reviewed. We hope to see an improvement in these two steps with the introduction of our in-house phlebotomy clinic.

**Conclusion:** Our findings show that the changes in process have had a positive impact regarding the reduction in time between blood tests being taken and the blood results being reviewed. We hope to see a continued improvement in this and all steps of the process with the introduction of our in-house phlebotomy clinic.

